# Evaluation of Patients With Cognitive Impairment Due to Suspected Idiopathic Normal-Pressure Hydrocephalus at Medical Centers for Dementia: A Nationwide Hospital-Based Survey in Japan

**DOI:** 10.3389/fneur.2022.810116

**Published:** 2022-05-27

**Authors:** Hiroaki Kazui, Mamoru Hashimoto, Shigetoshi Takeda, Yasuji Chiba, Tokiko Goto, Katsuhiro Fuchino

**Affiliations:** ^1^Department of Neuropsychiatry, Kochi Medical School, Kochi University, Nankoku, Japan; ^2^Department of Neuropsychiatry, Faculty of Medicine, Kindai University, Osakasayama, Japan; ^3^Seimou Hospital, Tomioka, Japan; ^4^Sanai Hospital, Noboribetsu, Japan; ^5^Akita Midorigaoka Hospital, Akita, Japan; ^6^Midorigaoka Hoyoen Hospital, Oita, Japan

**Keywords:** idiopathic normal-pressure hydrocephalus, medical centers for dementia, dementia specialists, evaluation, neurosurgeons, clinical guidelines, cerebrospinal fluid tap test, questionnaire survey

## Abstract

**Objective:**

Treatment of idiopathic normal-pressure hydrocephalus (iNPH) requires collaboration between dementia specialists and neurosurgeons. The role of dementia specialists is to differentiate patients with iNPH from patients with other dementia diseases and to determine if other dementia diseases are comorbid with iNPH. We conducted a nationwide hospital-based questionnaire survey on iNPH in medical centers for dementia (MCDs).

**Methods:**

We developed a questionnaire to assess how physicians in MCDs evaluate and treat patients with cognitive impairment due to suspected iNPH and the difficulties these physicians experience in the evaluation and treatment of patients. The questionnaire was sent to all 456 MCDs in Japan.

**Results:**

Questionnaires from 279 MCDs were returned to us (response rate: 61.2%). Patients underwent cognitive tests, evaluation of the triad symptoms of iNPH, and morphological neuroimaging examinations in 96.8, 77.8, and 98.2% of the MCDs, respectively. Patients with suspected iNPH were referred to other hospitals (e.g., hospitals with neurosurgery departments) from 78.9% of MCDs, and cerebrospinal fluid (CSF) tap test was performed in 44 MCDs (15.8%). iNPH guidelines (iNPHGLs) and disproportionately enlarged subarachnoid space hydrocephalus (DESH), a specific morphological finding, were used and known in 39.4% and 38% of MCDs, respectively. Logistic regression analysis with “Refer the patient to other hospitals (e.g., hospitals with neurosurgery departments) when iNPH is suspected.” as the response variable and (a) using the iNPHGLs, (b) knowledge of DESH, (c) confidence regarding DESH, (d) difficulty with performing brain magnetic resonance imaging, (e) knowledge of the methods of CSF tap test, (f) absence of physician who can perform lumbar puncture, and (g) experience of being told by neurosurgeons that referred patients are not indicated for shunt surgery as explanatory variables revealed that the last two factors were significant predictors of patient referral from MCDs to other hospitals.

**Conclusion:**

Sufficient differential or comorbid diagnosis using CSF tap test was performed in a few MCDs. Medical care for patients with iNPH in MCDs may be improved by having dementia specialists perform CSF tap tests and share the eligibility criteria for shunt surgery with neurosurgeons.

## Introduction

Normal-pressure hydrocephalus (NPH) is a syndrome that presents as cognitive impairment, gait disturbance, and urinary incontinence, known as the triad symptoms of NPH, in patients with enlarged ventricles under normal cerebrospinal fluid (CSF) pressure (1). NPH is now regarded as a syndrome that presents with balance disorders (2), motor abnormalities involving the upper limbs (3), and disturbances of the eye (4), in addition to its triad symptoms. Generally, NPH is classified as idiopathic NPH (iNPH), which has no identifiable causative antecedent disease, or secondary NPH, which develops after antecedent disease such as meningitis or subarachnoid hemorrhage. The estimated prevalence of iNPH in elderly Japanese and Swedish populations is 1.1 and 2.1%, respectively (5, 6). These figures indicate the worldwide prevalence of iNPH. In several studies, remarkable improvement in activities of daily living was reported in 69% (7), 69% (8), and 63% (9) of patients 1 year after undergoing shunt surgery for iNPH.

Secondary NPH occurs several months after the onset of the preceding disease; hence, it is not always necessary to differentiate it from other diseases. In contrast, iNPH is a slowly progressive disorder that requires differential diagnosis from other dementia diseases such as Alzheimer's disease (AD), dementia with Lewy bodies (DLB), and vascular dementia (VaD). In recent studies, it was reported that AD, DLB, and VaD are often comorbid with iNPH (10–12), and these comorbidities reduce the efficacy of shunt surgery (10, 11). In addition, iNPH is sometimes comorbid with schizophrenia (13), and patients with iNPH and prominent psychiatric symptoms are more likely to be admitted to psychiatric hospitals than to neurosurgical facilities (14). The prevalence of iNPH in patients with dementia as documented in memory disorder clinics is 15% (15), highlighting that many patients with iNPH visit dementia facilities. Thus, dementia specialists, including psychiatrists, should be involved in the differentiation of iNPH from other dementia/psychiatric diseases and in the diagnosis of the comorbidities of iNPH.

The elderly population is growing rapidly in Japan. It is estimated that the number of people with dementia will exceed 7 million by 2025, and that one out of every five people aged 65 years or older in Japan will experience dementia. In 2008, medical centers for dementia (MCDs) were established as part of a new national health program against dementia to ensure that Japanese citizens receive appropriate medical care for dementia regardless of where they live. The MCDs were established at medical institutions designated by the governors of the 47 prefectures in Japan or the mayors of 20 designated cities. The aim was to have at least one MCD in each secondary medical care area in Japan for a total of 500 centers nationwide. MCDs contribute to the provision of the following (16): (1) special medical consultation; (2) differential diagnosis and early intervention; (3) medical treatment for the acute stage of behavioral and psychological symptoms of dementia and concurrent medical conditions; (4) education for general practitioners and other community professionals; (5) network meetings for the establishment of cooperation among medical facilities and collaboration between medical facilities and nursing care facilities; and (6) information regarding dementia to the public. There are three types of MCDs: core-type, regional-type, and collaborative-type MCDs. Regional-type MCDs are the most common type, and psychiatric hospitals are often designated as regional-type MCDs. Core-type MCDs include university hospitals and general hospitals, and the special role of core-type MCDs is to provide training of human resources for the evaluation and treatment of patients with dementia. Collaborative-type MCDs refer to clinics, and the special role of these MCDs is community-based collaboration with relevant institutions. The number of MCDs has been increasing, reaching 456 in 2019; consequently, all patients with dementia in Japan can now receive the treatment and care they need.

In the treatment of iNPH, collaboration between dementia specialists and neurosurgeons is necessary. However, a small-scale survey we conducted in 2013 revealed that physicians in many MCDs do not perform CSF tap test and that physicians in more than half the MCDs find that “patients are referred to neurosurgeons, but the neurosurgeons said the patients are not indicated for shunt surgery” (17). These findings indicate that there may be room for improvement regarding the clinical practice of physicians in MCDs for patients with iNPH and regarding collaboration between physicians and neurosurgeons. However, there have been no large-scale studies evaluating the actual status of examinations and treatment of patients with iNPH in specialized facilities for patients with dementia. Therefore, we conducted a nationwide hospital-based questionnaire survey to evaluate how patients with cognitive impairment due to suspected iNPH are examined and treated in MCDs 6 years after our previous preliminary survey.

## Materials and Methods

### Contents of the Questionnaire and Background

The contents of the questionnaire used in this study are based on the diagnostic criteria for iNPH in the second edition of the Japanese iNPH guidelines (iNPHGLs) (5). In the Japanese iNPHGLs, iNPH is categorized into three diagnostic levels: preoperatively “possible,” preoperatively “probable,” and postoperatively “definite.” A diagnosis of a possible iNPH is made if all the following five criteria are met: (1) development of symptoms in the 60's or when older; (2) the presence of more than one of the clinical triad symptoms, namely cognitive impairment, gait disturbance, and urinary incontinence; (3) ventricular enlargement (Evans index > 0.3) on brain computed tomography (CT) or magnetic resonance imaging (MRI); (4) the abovementioned clinical symptoms cannot be completely explained by other neurological or non-neurological diseases; and (5) the absence of prior diseases that may cause ventricular dilation. A diagnosis of probable iNPH is made if all the following three criteria are met: (1) the requirements for possible iNPH are met; (2) CSF pressure of ≤200 mmH_2_O and normal CSF content; and (3) one of the following three investigational features: (a) improvement in one or more of the triad symptoms following lumbar puncture performed to temporarily decrease the volume of CSF (CSF tap test); (b) improvement in one or more of the triad symptoms following CSF drainage test; or (c) the neuroimaging feature termed “disproportionately enlarged subarachnoid space hydrocephalus (DESH),” i.e., narrowing of the sulci and subarachnoid spaces over the high convexity/midline surface with ventricular enlargement (7), under the presence of gait disturbance. In clinical settings in Japan, a diagnosis of probable iNPH is made when one or more of the triad symptoms improve after the CSF tap test in patients with DESH. A probable iNPH patient is indicated for shunt surgery and is therefore referred to a hospital where neurosurgery is performed. Definite iNPH is defined as a patient for whom the symptoms improve after shunt surgery. The most important difference between the diagnostic criteria of the Japanese and American-European iNPHGLs (18) is the emphasis on DESH findings in the Japanese iNPHGLs.

We administered our questionnaire to assess how physicians in MCDs evaluate and treat patients with cognitive impairment due to suspected iNPH. We inquired about iNPHGLs used in MCDs and about difficulties encountered in the treatment of iNPH. We also included a question to assess the knowledge of physicians in MCDs regarding DESH (7). DESH is an important finding as it is useful for differentiating iNPH from AD and VaD (19), and it has a high positive predictive value in identifying shunt-responsive patients with iNPH (20). The questionnaire is shown in the Supplementary Material.

### Survey Procedure

The study period was from June 7, 2019 to March 31, 2020. On October 25, 2019, the questionnaire was sent to all 456 MCDs in Japan, which include 16 core-type, 367 regional-type, and 73 collaborative-type MCDs. The questionnaire was to be completed by the head of each MCD or by a physician currently working at the MCD. The deadline for returning the questionnaire was November 11, 2019. Both the participants in this study and the patients that they treated were believed to mostly be Japanese.

The study was conducted in accordance with the Declaration of Helsinki (2013) of the World Medical Association and the guidelines of the ethics committee of the Japan Psychiatric Hospitals Association.

### Analyses

We calculated the percentages of MCDs that answered “Yes” to each of the items in the questionnaire. Using Fisher's exact test, we compared the ratio of “Yes” answers between the three types of MCDs. In addition, logistic regression analysis was performed to determine factors that influence referral from MCDs to other hospitals (e.g., hospitals with neurosurgery departments) when iNPH is suspected. The response variable was “Refer the patient to other hospitals (e.g., hospitals with neurosurgery departments) when iNPH is suspected.” The explanatory variables were (a) I use iNPHGLs; (b) I am familiar with DESH; (c) “I am not confident in my assessment of DESH on brain MRI;” (d) “Brain MRI cannot be performed;” (e) “No physician who can perform lumbar puncture is available;” (f) “I have no knowledge of the methods or criteria for evaluating clinical symptoms in CSF tap test;” and (g) “Patients are referred to neurosurgeons, but the neurosurgeons said that the patients are not indicated for shunt surgery.” Odds ratios were calculated for all explanatory variables. Statistical analyses were performed using SPSS version 27, and significance level was set at *p* < 0.05.

## Results

### Characteristics of Participating MCDs

**Figure 1:**
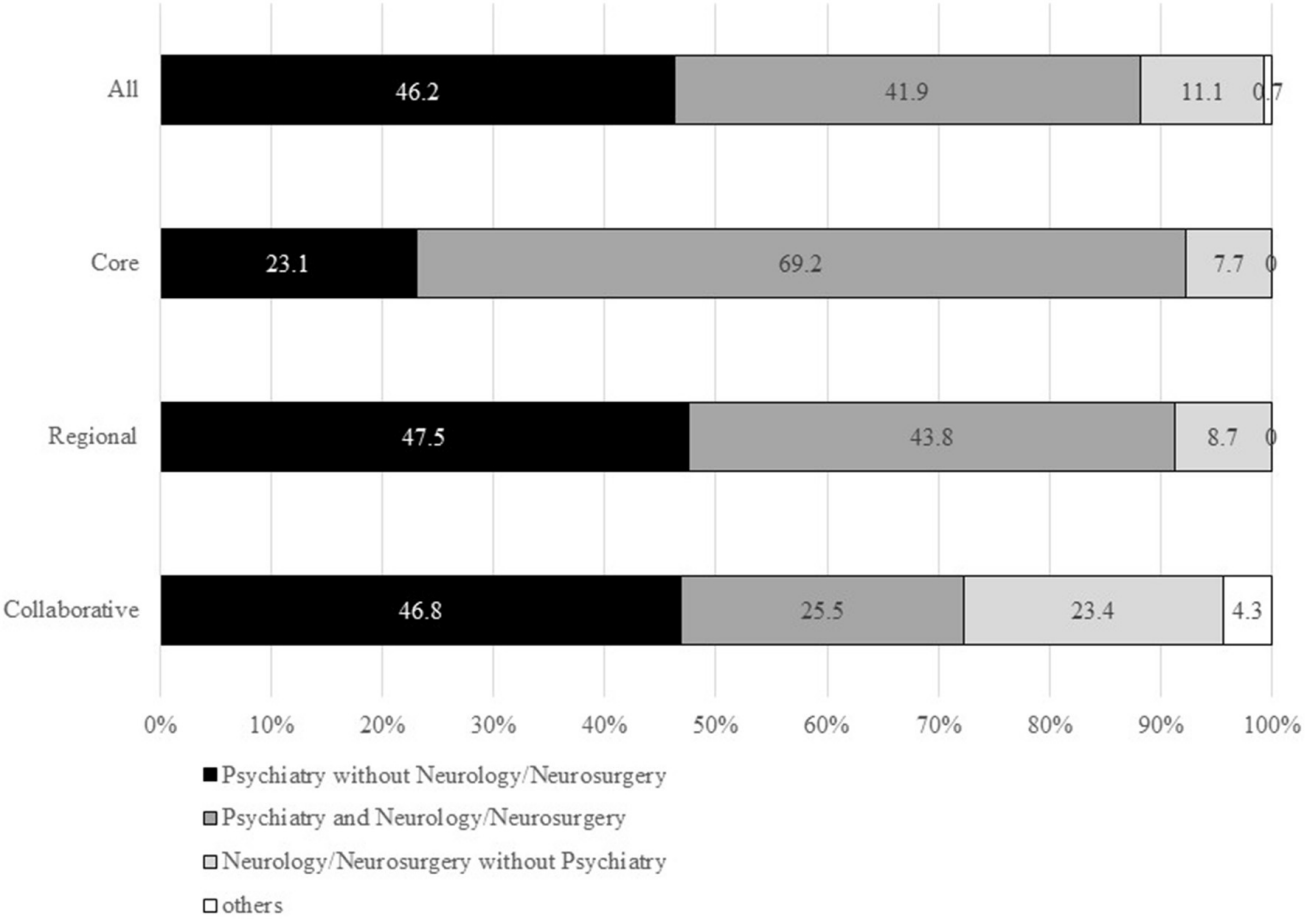
Percentages of departments in MCDs. Department of internal medicine was not included in this figure. MCD, medical center for dementia.

Of the 456 questionnaires sent, 279 were returned to us (response rate: 61.2%). Of the 279 responders, 13 (4.7%) were core-type MCDs, 219 (78.5%) were regional-type MCDs, and 47 (16.8%) were collaborative-type MCDs. The response rates by MCD type were 81.3% (13/16) for core-type MCDs, 59.7% (219/367) for regional-type MCDs, and 64.4% (47/73) for collaborative-type MCDs. Regarding departments in the 279 MCDs, 246 (88.2%) had a psychiatry department, 187 (67.0%) had an internal medicine department, 123 (44.1%) had a neurology department, and 66 (23.7%) had a neurosurgery department. The combination of psychiatry and neurology/neurosurgery and the psychiatry without neurology/neurosurgery were similar in percentage in all MCDs (Figure 1). Given that most core-type MCDs are university hospitals or general hospitals, the percentage of core-type MCDs with the psychiatry and neurology/neurosurgery departments was found to be 69.2%. Collaborative-type MCDs have a higher percentage of centers with a neurology/neurosurgery department but without a psychiatry department than core-type or regional-type MCDs.

Regarding neuroimaging examinations, brain CT was performed in 269 MCDs (96.4%), either at the facility or at collaborating facilities. Brain MRI scans were performed in 161 MCDs (57.7%), including 11 core-type MCDs (84.6%), 119 regional-type MCDs (54.3%), and 31 collaborative-type MCDs (66.0%). CSF examination was performed in 94 MCDs (33.7%), including 10 core-type MCDs (76.9%), 72 regional-type MCDs (33.0%), and 12 collaborative-type MCDs (25.5%).

### Results of Questionnaire Survey

#### Evaluation and Treatment of Patients With Suspected iNPH

**Figure 2:**
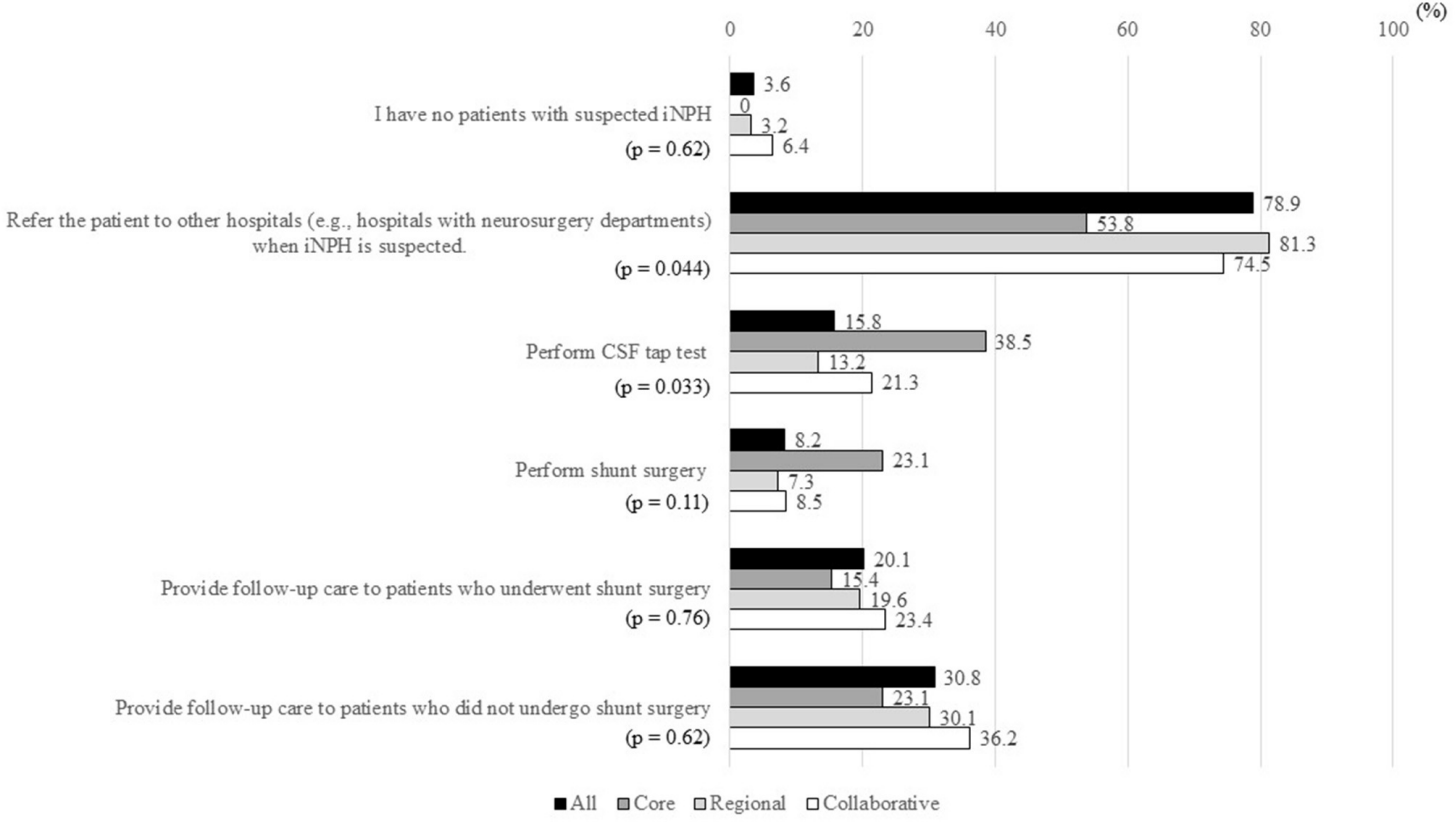
Percentages of MCDs that answered “Yes” to items in response to the question “How do you evaluate and treat patients with suspected iNPH?”. MCD, medical center for dementia; iNPH, idiopathic normal-pressure hydrocephalus; CSF, cerebrospinal fluid. *The ratio of “Yes” answers were compared between the three types of MCDs using Fisher's exact test.

Ten (3.6%) of the 279 MCDs had no patients with suspected iNPH (Figure 2). Patients with suspected iNPH were referred to other hospitals (e.g., hospitals with neurosurgery departments) from 220 MCDs (78.9%), CSF tap test was performed in 44 MCDs (15.8%), and shunt surgery was performed in 23 MCDs (8.2%). Follow-up care for patients with suspected iNPH who underwent shunt surgery and for patients with suspected iNPH who did not undergo shunt surgery was performed in 56 MCDs (20.1%) and 86 MCDs (30.8%), respectively.

Significant differences in the items “Refer the patient to other hospitals (e.g., hospitals with neurosurgery departments) when iNPH is suspected” and “Perform CSF tap test” were observed between the three types of MCDs (*p* = 0.044 and *p* = 0.033, respectively). The rate of the former item was lower and the rate of the latter item was higher in core-type MCDs than in regional-type or collaborative-type MCDs.

#### Examinations of Patients With Suspected iNPH

**Figure 3:**
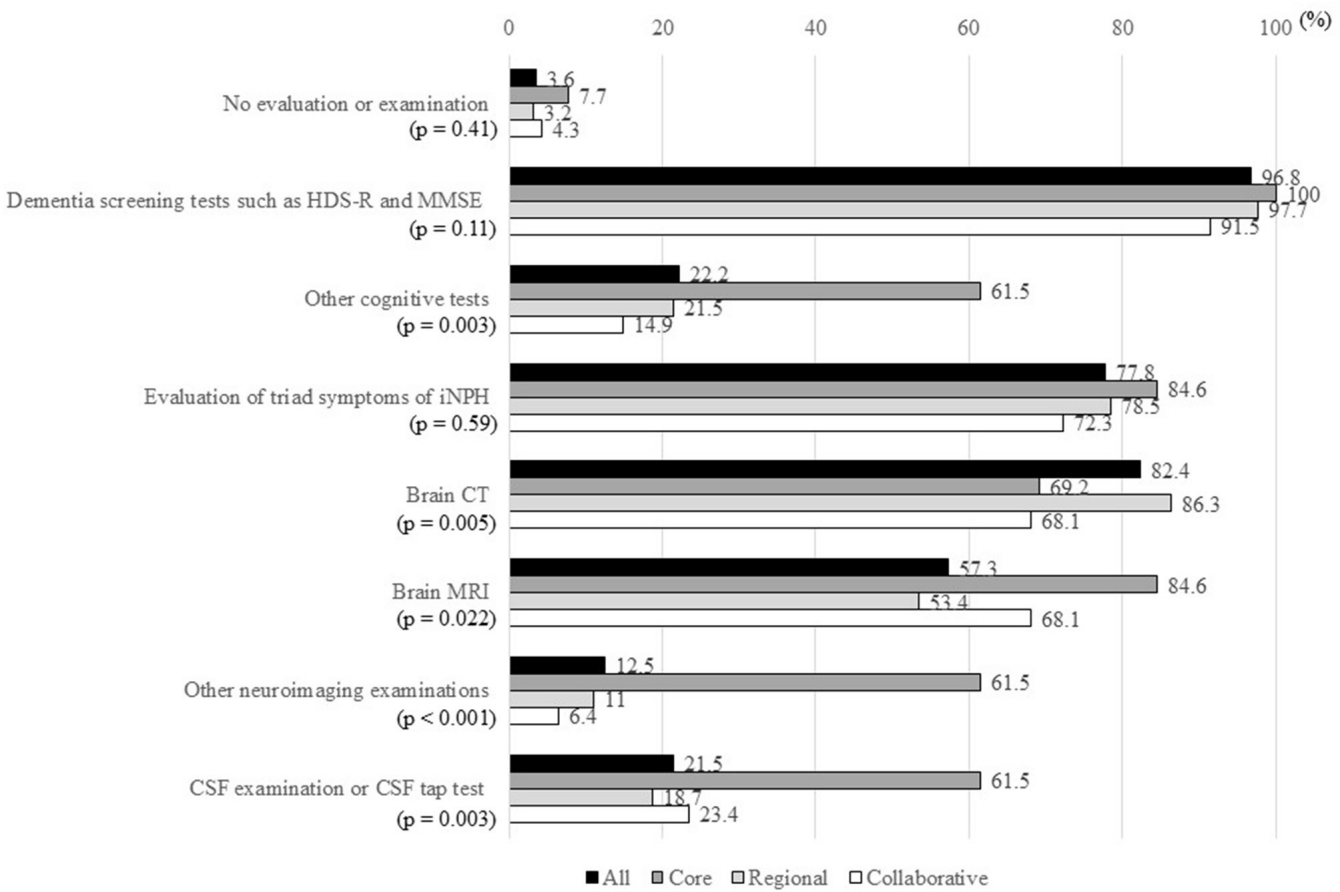
Percentages of MCDs that answered “Yes” to items in response to the question “How do you examine patients with suspected iNPH?”. MCD, medical center for dementia; HDS-R, Hasegawa Dementia Scale-Revised; MMSE, Mini Mental State Examination; iNPH, idiopathic normal-pressure hydrocephalus; CT, computed tomography; MRI, magnetic resonance imaging; CSF, cerebrospinal fluid. *The ratio of “Yes” answers were compared between the three types of MCDs using Fisher's exact test.

Ten MCDs (3.6%) did not perform any examinations for patients with suspected iNPH (Figure 3). Cognitive screening tests, such as assessment using the Hasegawa Dementia Scale-Revised and Mini Mental State Examination, were performed in 270 of the 279 MCDs (96.8%). Other cognitive tests, including Frontal Assessment Battery, Alzheimer's Disease Assessment Scale, and Rivermead Behavioral Memory Test, were performed in 62 MCDs (22.2%). Evaluation of the triad symptoms of iNPH was performed in 217 MCDs (77.8%), and brain CT or MRI was performed in 274 MCDs (98.2%). Other neuroimaging examinations, most commonly cerebral perfusion single-photon emission CT, were performed in 35 MCDs (12.5%). CSF examination or CSF tap test was performed in 60 MCDs (21.5%).

There were significant differences in other cognitive tests, brain CT, brain MRI, other neuroimaging examinations, and CSF examination/CSF tap test between the three types of MCDs. The percentages of all items, except “Perform brain CT,” were higher in core-type MCDs than in regional-type or collaborative-type MCDs.

#### Use of iNPHGLs

**Figure 4:**
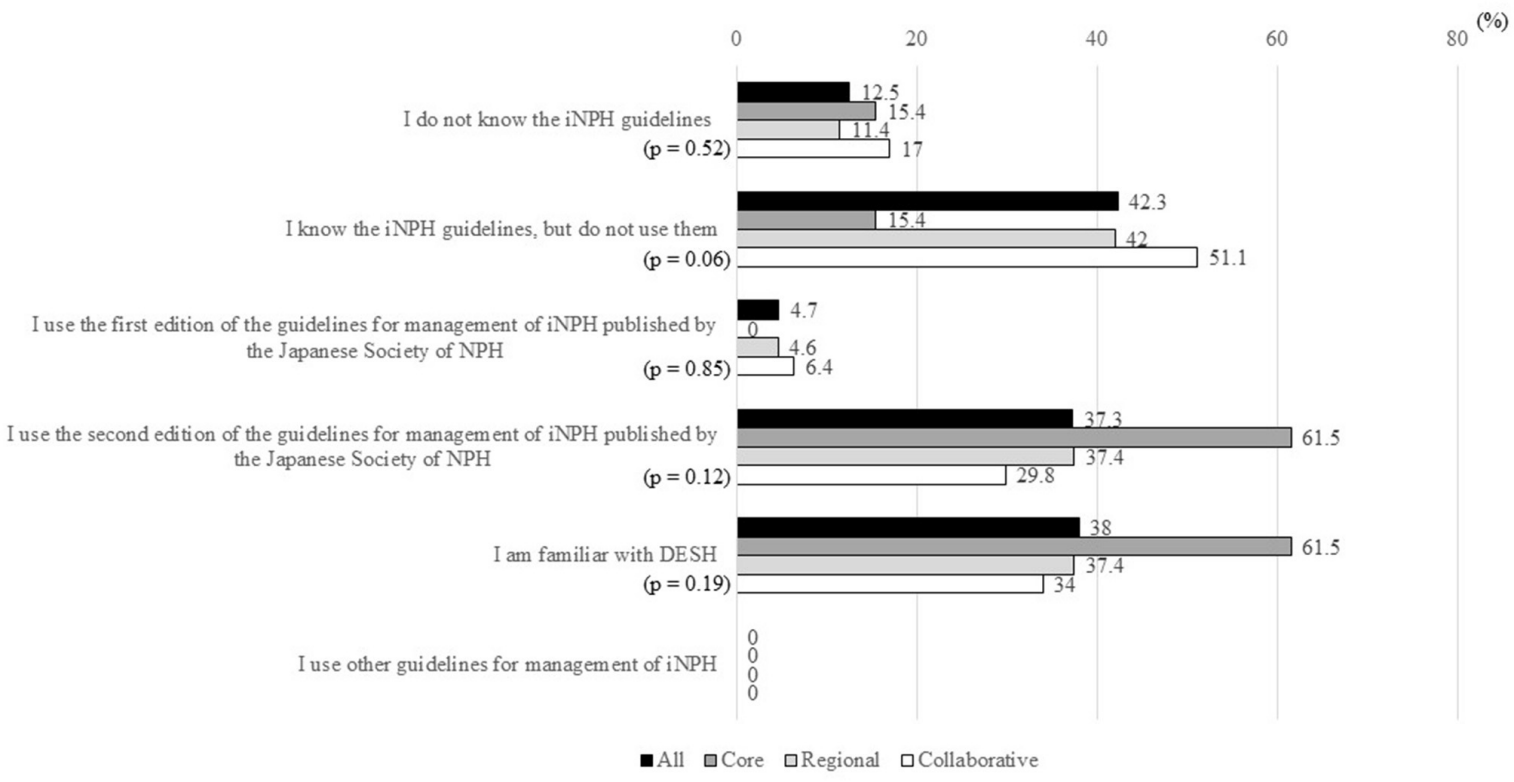
Percentages of MCDs that answered “Yes” to items in response to the question “Do you know and use the guidelines for the management of iNPH?”. MCD, medical center for dementia; iNPH, idiopathic normal-pressure hydrocephalus; NPH, normal-pressure hydrocephalus; DESH, disproportionately enlarged subarachnoid space hydrocephalus. *The ratio of “Yes” answers were compared between the three types of MCDs using Fisher's exact test.

In 35 MCDs (12.5%), iNPHGLs were not known, and in 118 MCDs (42.3%), iNPHGLs were known but were not used (Figure 4). The first edition of the Japanese iNPHGLs (21) was used in 13 MCDs (4.7%), while the second edition of the Japanese iNPHGLs (5) was used in 104 MCDs (37.3%). With both editions of the Japanese iNPHGLs used in 7 MCDs, the total number (and percentage) of MCDs that use the iNPHGLs was 110 (39.4%). No MCDs reported using iNPHGLs other than the Japanese iNPHGLs. There was familiarity with DESH in 106 MCDs (38%), and no statistically significant differences in the use of iNPHGLs were observed between the three types of MCDs.

#### Difficulties in Evaluation and Treatment of Patients With Suspected iNPH

**Figure 5:**
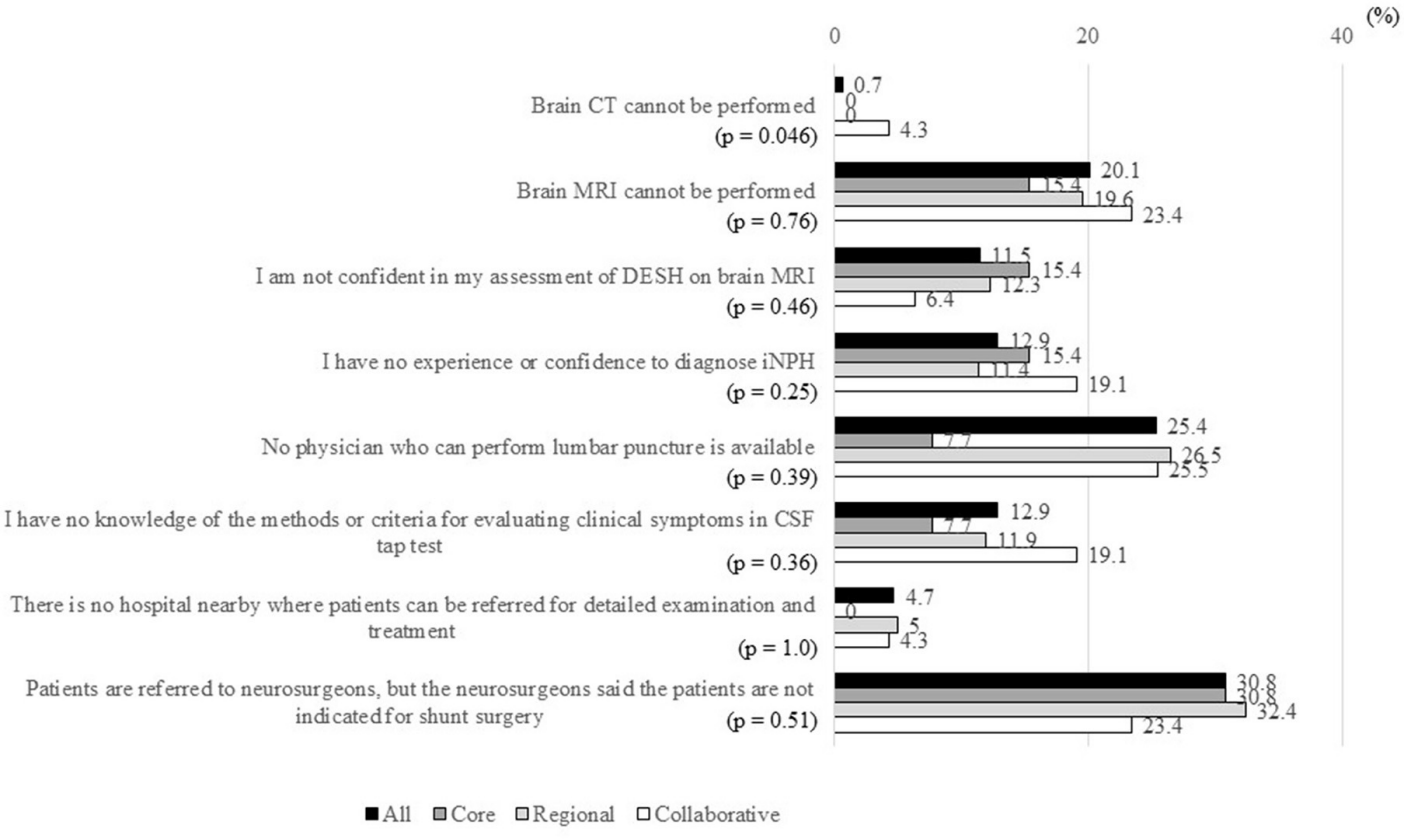
Percentages of MCDs that answered “Yes” to items in response to the question “What are the difficulties in the evaluation and treatment of patients with suspected iNPH?”. MCD, medical center for dementia; CT, computed tomography; MRI, magnetic resonance imaging; CSF, cerebrospinal fluid; DESH, disproportionately enlarged subarachnoid space hydrocephalus; iNPH, idiopathic normal-pressure hydrocephalus; CSF, cerebrospinal fluid. *The ratio of “Yes” answers were compared between the three types of MCDs using Fisher's exact test.

The item “Patients are referred to neurosurgeons, but the neurosurgeons said the patients are not indicated for shunt surgery” was the most common difficulty in MCDs, affecting 86 MCDs (30.8%; Figure 5). The second most common difficulty was the item “No physician who can perform lumbar puncture is available,” affecting 71 MCDs (25.4%). About 12% of MCDs answered “Yes” to the items “I am not confident in my assessment of DESH on brain MRI,” “I have no experience or confidence to diagnose iNPH,” and “I have no knowledge of the methods or criteria for evaluating clinical symptoms in CSF tap test.”

Except for the item “Brain CT cannot be performed,” significant differences were not observed in any item regarding difficulties in the evaluation and treatment of patients with iNPH between the three types of MCDs. The item “Brain CT cannot be performed” was a problem only in 4.3% of collaborative-type MCDs.

#### Intentions for Examination and Treatment for Patients With Cognitive Impairment Due to Suspected iNPH at MCDs

**Figure 6:**
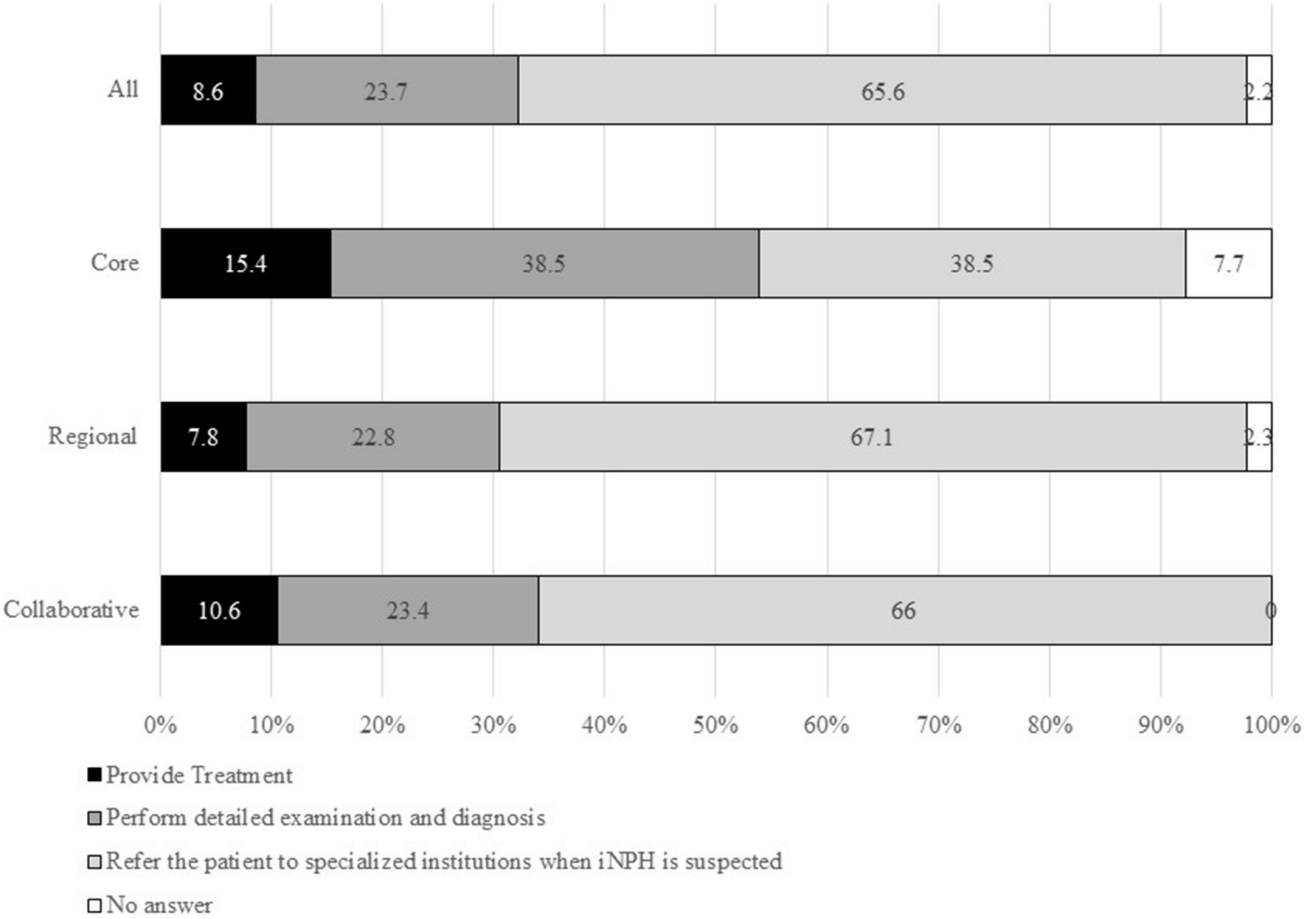
Percentages of “Yes” in answer to “To what extent do you think MCDs should evaluate and treat patients with cognitive impairment due to suspected iNPH?”.

Overall, 183 MCDs (65.6%) responded “Yes” to “Refer the patient to specialized institutions when iNPH is suspected,” 66 MCDs (23.7%) responded “Yes” to “Perform detailed examination and diagnosis,” and 24 MCDs (8.6%) replied “Yes” to “Provide treatment” (Figure 6). The percentages of the last two items were slightly higher in core-type MCDs than in regional-type or collaborative-type MCDs.

### Predictors of Referral From MCDs to Other Hospitals When iNPH Is Suspected

Logistic regression analysis revealed that “No physician who can perform lumbar puncture is available” and “Patients are referred to neurosurgeons, but the neurosurgeons said the patients are not indicated for shunt surgery” were significant predictors of patient referral from MCDs to other hospitals (e.g., hospitals with neurosurgery departments) when iNPH is suspected (Table 1).

**Table 1:** Predictors of patient referral from MCDs to other hospitals when iNPH is suspected.

**Explanatory variables**	**B**	**SE**	**Waldχ2**	*p*-Value^*^	**OR**	**95%CI**
I used guidelines for management of iNPH.	−0.636	0.369	2.959	0.085	0.530	0.257–1.093
I am familiar with DESH.	−0.082	0.378	0.047	0.828	0.921	0.440–1.932
I am not confident in my assessment of DESH on brain MRI.	0.735	0.817	0.809	0.368	2.085	0.421–10.333
Brain MRI cannot be performed.	0.731	0.535	1.869	0.172	2.077	0.728–5.921
No physician who can perform lumbar puncture is available.	1.370	0.562	5.931	0.015	3.934	1.307–11.848
I have no knowledge of the methods or criteria for evaluating clinical symptoms in CSF tap test.	0.651	0.794	0.672	0.412	1.917	0.404–9.094
Patients are referred to neurosurgeons, but the neurosurgeons said the patients are not indicated for shunt surgery.	1.358	0.426	10.170	0.001	3.888	1.688–8.958

* *Logistic regression analysis*.

*B, partial regression coefficient; SE, standard error; Wald χ2, Wald χ2 statistic; OR, odds ratio; 95%CI, 95% confidence interval*.

## Discussion

We conducted a nationwide hospital-based questionnaire survey to determine how patients with cognitive impairment due to suspected iNPH, a treatable dementia, were evaluated and treated in MCDs in Japan. This is the first study to investigate the actual status of examination and treatment of patients with iNPH in specialized facilities for patients with dementia. This survey revealed that most MCDs cater to patients with cognitive impairment due to suspected iNPH and that sufficient differential or comorbid diagnosis using the CSF tap test was performed at a few MCDs. Most MCDs referred patients to other hospitals (e.g., hospitals with neurosurgery departments) when iNPH was suspected. Two hundred and seventy-nine MCDs in Japan responded to the questionnaire (response rate: 61.2%). The response rates of the three types of MCDs were around 60% or more, and the results of this study can be considered reflective of the current situation in MCDs in Japan. As regional-type MCDs are originally the most common type of MCDs, the overall results of this study are most reflective of the situation in regional-type MCDs. In terms of departments, most MCDs have a psychiatry department and a department of internal medicine. About 40% of MCDs have a neurology department, and about 20% of MCDs have a neurosurgery department.

This survey revealed that a small percentage (3.6%) of MCDs do not attend to patients with suspected iNPH, meaning that most MCDs cater to these patients. Patients with suspected iNPH underwent cognitive screening tests, evaluation of the triad symptoms of iNPH, and morphological neuroimaging examinations in most MCDs. Thus, they were diagnosed with possible iNPH in MCDs based on the presence of the triad symptoms and ventricular enlargement. However, patients with AD, DLB, or VaD may also meet the diagnostic criteria for possible iNPH. Therefore, patients with possible iNPH should be differentiated between those with probable iNPH or those with other dementia diseases. In this survey, CSF tap test was performed in only 15.8% of MCDs, and 78.9% of MCDs referred patients to other hospitals (e.g., hospitals with neurosurgery departments) when iNPH was suspected, indicating that probable iNPH was not diagnosed in most MCDs. Since all neurosurgeons are not skilled in the differential diagnosis of dementia, all patients with possible iNPH do not receive appropriate differential diagnosis in neurosurgical facilities after referral from MCDs. Before considering shunting, it is important that patients with iNPH who also have AD or DLB and the families of these patients understand that another disease is comorbid with iNPH. Hence, it is appropriate that physicians in MCDs refer patients to neurosurgery for shunting after detailed differential and comorbid diagnoses, including CSF tap test.

In this study, we determined the predictors of patient referral from MCDs to other hospitals (e.g., hospitals with neurosurgery departments) when iNPH is suspected. Our results show that “No physician who can perform lumbar puncture is available” is a significant predictor of patient referral. This finding is logical given that CSF tap test cannot be performed without lumbar puncture, which requires training. Lumbar puncture is an important procedure for physicians in MCDs because it is often used to differentiate meningitis from dementia (22, 23). In addition, amyloid-β, phosphorylated tau, and total tau levels in CSF are useful for differentiating AD from other dementia diseases (24). CSF biomarkers also contribute to differentiating iNPH from other diseases (25–28), making a differential diagnosis of mixed cases (29), and predicting the improvement of clinical symptoms after shunt surgery in patients with iNPH (30, 31). In this survey, it was found that 23.7% of MCDs responded with “Perform detailed examination and diagnosis” to the question “To what extent do you think MCDs should evaluate and treat patients with cognitive impairment due to suspected iNPH?”. Furthermore, in this survey, CSF examination was conducted in 33.7% of MCDs, which is higher than the percentage of MCDs where CSF tap test was performed (15.8%). Thus, in the near future, the number of MCDs where CSF tap test is performed is expected to increase.

The item “Patients are referred to neurosurgeons, but the neurosurgeons said the patients are not indicated for shunt surgery” was found to be another predictive factor of referral of patients with suspected iNPH to other hospitals. Physicians in MCDs who referred patients with iNPH to the neurosurgery department after performing detailed evaluations, such as CSF tap test, may feel that it is useless to examine patients, causing the physicians to immediately refer patients to the neurosurgery department. On the other hand, physicians in MCDs who referred patients with iNPH without performing detailed examinations may decide against referral to the neurosurgery department but decide to refer these patients to specialized hospitals, such as university hospitals, where detailed examinations, including CSF tap test, are performed. We were unable to verify these assumptions because the exact departments/hospitals patients were referred to are not known.

Regarding the difference in understanding between neurosurgeons and physicians pertaining to the eligibility of patients with suspected iNPH for shunt surgery, neurosurgeons may exercise caution because shunt surgery improves cognitive impairment to a lesser degree than gait disturbance (32), because patients with severe preoperative cognitive impairment are likely to have residual cognitive impairment after shunt surgery (33), and because patients who visit dementia centers and psychiatric institutions are known to have more severe cognitive impairment than patients who visit neurosurgery departments (14). In addition, remarkable cognitive impairment in patients with iNPH may be due to other comorbid dementia diseases that can reduce cognitive improvement following shunt surgery (10, 11). Furthermore, the low proportion of MCDs where physicians are familiar with DESH in this study indicates that many physicians who are unfamiliar with DESH referred patients with suspected iNPH to neurosurgeons. Use of the iNPHGLs may help reduce the difference in understanding pertaining to the eligibility of patients with suspected iNPH for shunt surgery between neurosurgeons and physicians in MCDs. This is attributable to the fact that the results of the logistic regression analysis in this study showed that MCDs using the iNPHGLs had a lower rate of referral of patients to other hospitals (e.g., hospitals with neurosurgery departments) when iNPH was suspected (*p* = 0.085). The iNPHGLs may help physicians in MCDs understand that the main roles of the MCDs include the differential and comorbid diagnoses for patients with suspected iNPH. The number of MCDs where CSF tap test is performed is expected to increase, as we mentioned above. If the physicians in MCDs refer the iNPH patients with DESH and whose symptoms improve on the CSF tap test to neurosurgeons, neurosurgeons will be likely to report that there is an indication for shunt surgery for patients with cognitive impairment at MCDs. Moreover, a consensus on the eligibility of patients with iNPH who have remarkable cognitive impairment and/or comorbidity for shunt surgery can be reached via direct communication between physicians in MCDs and neurosurgeons.

It is important that the contents of the questionnaire used in this study are based on the diagnostic criteria for iNPH in the second edition of the Japanese iNPHGLs (5), which differ from the diagnostic criteria for iNPH in the American-European iNPHGLs (18). The Japanese iNPHGLs include an age of onset older than 60 years and any one of the triad symptoms, whereas the American-European iNPHGLs include an age of onset over 40 years and mandatory gait or balance disturbance, in addition to one of the other two symptoms of the triad. Thus, an increasing number of patients without gait disturbance are likely diagnosed with iNPH in Japan compared with European and North American countries. Regarding neuroimaging findings, the Japanese iNPHGLs emphasize the DESH features; in contrast, in the American-European iNPHGLs, ventriculomegaly, a narrow callosal angle, enlargement of the temporal horns, and periventricular signal changes not attributable to ischemic changes or demyelination are considered important (18, 34, 35). Obtaining a diagnosis of “probable iNPH” is three times more likely as per the American-European iNPHGLs compared with the Japanese iNPHGLs (35). The number of iNPH patients with DESH may be as low as one third of all iNPH patients, and it is thus possible that fewer patients are being actively treated in Japan than in European and North American countries. Patients with iNPH who have DESH may be the focus of treatment for MCD physicians who are not experts in iNPH because patients with DESH are easier to detect and more likely to benefit from shunt surgery (20).

The results of this study may be affected by the current status of treatment for iNPH in Japan, which differs from that in European and North American countries. First, Japan has many neurosurgery hospitals, which tend to be smaller and thus have smaller referral areas than hospitals in European and North American countries. In two studies conducted in Japan, 87 patients with iNPH underwent shunt surgery in 20 centers (approximately four patients per center) (9) and 1,608 patients with iNPH underwent shunt surgery in 289 facilities (approximately six patients per center) (36). In contrast, in a European multicenter study, 140 patients with iNPH underwent shunt surgery in 13 centers in 1 year (approximately 11 patients per center) (8), and in a study conducted in North America, 151 patients were examined for 11 years in one center (~14 patients per year per center) (37). These findings suggest that neurosurgeons in Japan have less experience in treating iNPH patients compared with those in Europe and North America. Thus, some neurosurgeons in Japan may be unfamiliar with the diagnostic criteria of the Japanese iNPHGLs. Second, although ventriculoperitoneal (VP) shunt surgery is the most common worldwide, lumboperitoneal (LP) shunt surgery is the most common shunt surgery for iNPH in Japan (36). An advantage of LP shunt surgery is that patients and their caregivers are less likely to oppose to this procedure than VP shunt surgery because the LP approach can help circumventing cranial surgery. Thus, there may be more patients with iNPH requesting shunt surgery in Japan than in other countries. Third, there may be a considerable number of iNPH patients with comorbid dementia diseases other than iNPH, such as Alzheimer's disease, because of the aging population of Japan.

In 2013, we conducted a small-scale questionnaire survey similar to this survey to evaluate how patients with iNPH are examined in MCDs (17). Ninety-eight of 205 MCDs responded to our previous questionnaire survey (response rate: 48%). We compared the results of this 2019 survey to those of our 2013 survey. The second edition of the Japanese iNPHGLs was published in 2011 (5). Thus, our two surveys were conducted 2 and 8 years after the publication of the revised iNPHGLs, respectively. The response rate and amount of data in this study were greater than those in the previous study. Compared to the 2013 survey, fewer physicians in this study responded in the affirmative to the items “I have no patients with suspected iNPH” (3.6% in 2019 vs. 8% in 2013) and “Patients are referred to neurosurgeons, but the neurosurgeons said the patients are not indicated for shunt surgery” (30.8% in 2019 vs. 56% in 2013). Further, in this survey, more physicians in MCDs answered “Yes” to the item “Provide follow-up care to patients who did not undergo shunt surgery” (30.8% in 2019 vs. 2% in 2013). These results suggest increased frequency of examinations and follow-up care of patients with iNPH in MCDs and less difference in understanding between neurosurgeons and physicians pertaining to the eligibility of patients with suspected iNPH for shunt surgery over the past 6 years. However, there was a reduction in the use of the iNPHGLs (39.4% in 2019 vs. 45.0% in 2013) and in the knowledge of DESH (38.0% in 2019 vs. 54.0% in 2013). The 2013 survey was conducted by the organizers of the 15th annual meeting of Japanese Society for NPH. Thus, more MCDs where diagnosis and treatment of iNPH are performed may have participated in the previous survey than in this survey. These results suggest that it is necessary to further educate physicians in MCDs on the iNPHGLs and on DESH to ensure accurate diagnosis of iNPH and to improve collaboration with neurosurgeons.

Comparison of results between the three types of MCDs revealed little difference between regional-type and collaborative-type MCDs. The percentages of administration of cognitive tests other than screening tests, brain MRI and other neuroimaging examinations, and CSF examination/CSF tap test were higher in core-type MCDs than in regional-type or collaborative-type MCDs. Further, patient referral to other hospitals (e.g., hospitals with neurosurgery departments) when iNPH was suspected was less likely in core-type MCDs than in regional-type or collaborative-type MCDs. These results are considered logical since university hospitals and general hospitals constitute core-type MCDs. However, there were no significant differences in the use of the iNPHGLs and knowledge of DESH between the three types of MCDs. Since the special role of core-type MCDs is to train human resources to treat dementia, including iNPH, it is important that the percentages of administration of CSF tap test, use of the iNPHGLs, and knowledge of DESH in core-type MCDs increase.

This study has a few limitations. First, the results were not confirmed with actual figures. Second, there may be a type 2 error in the results of the comparison between the three types of MCDs due to the small number of core-type MCDs. Third, the departments of the physicians who completed the questionnaire were not determined. Fourth, serial CSF removal in older adults with iNPH may be an alternative treatment option for those patients who refuse or have a contraindication to shunt surgery (38). However, we did not include this treatment option in our questionnaire because serial CSF removal is rarely performed in Japan. Fifth, our questionnaire might not be structured to cover all the multidisciplinary aspects of the diagnostic and therapeutic work out of iNPH. Finally, the response rate to the questionnaire survey was not high.

The outcome of shunt surgery may be improved if differential and comorbid diagnoses of iNPH are performed in more MCDs before patient referral to the neurosurgery department. Creating a collaborative relationship between physicians in MCDs and neurosurgeons will be crucial to the provision of necessary multidisciplinary care to effectively evaluate, diagnose, and treat patients with iNPH.

## Data Availability Statement

The raw data supporting the conclusions of this article will be made available by the authors, without undue reservation.

## Ethics Statement

The studies involving human participants were reviewed and approved by the Ethics Committee of the Japan Psychiatric Hospitals Association. Written informed consent for participation was not required for this study in accordance with the national legislation and the institutional requirements.

## Author Contributions

HK, MH, ST, YC, TG, and KF designed the study and supervised the data collection. HK was responsible for the statistical design of the study and for performing the statistical analysis and wrote the first manuscript draft. All authors were involved in the interpretation or presentation of the data, reviewed and revised the initial draft and subsequent versions of the report, and approved the submitted version.

## Funding

This work was supported in part by a Research project on health and welfare promotion for the elderly from the Japanese Ministry of Health, Labor and Welfare (Project No. 2019-93) and in part Grant-in-Aid for Scientific Research (B) (No. JP19H03585).

## Conflict of Interest

HK has received speaker's honoraria from Integra Japan K. K. The remaining authors declare that the research was conducted in the absence of any commercial or financial relationships that could be construed as a potential conflict of interest.

## Publisher's Note

All claims expressed in this article are solely those of the authors and do not necessarily represent those of their affiliated organizations, or those of the publisher, the editors and the reviewers. Any product that may be evaluated in this article, or claim that may be made by its manufacturer, is not guaranteed or endorsed by the publisher.
