# Supplementary material for: Evaluation of Patients With Cognitive Impairment Due to Suspected Idiopathic Normal-Pressure Hydrocephalus at Medical Centers for Dementia: A Nationwide Hospital-Based Survey in Japan
Source: Front Neurol. 2022 May 27;13:810116. doi: 10.3389/fneur.2022.810116 (PMC9184737; doi:10.3389/fneur.2022.810116)
Supplement: Supplementary file 1 [file Data_Sheet_1.PDF]

Appendix. Questionnaire for evaluating patients with cognitive impairment due to suspected idiopathic normal-pressure hydrocephalus (iNPH) in medical centers for dementia (MCDs)

(1) How do you evaluate and treat patients with suspected iNPH? Please check all items that apply.

1. I have no patients with suspected iNPH.
2. Refer the patient to other hospitals (e.g., hospitals with neurosurgery departments) when iNPH is suspected.
3. Perform cerebrospinal fluid (CSF) tap test.
4. Perform shunt surgery.
5. Provide follow-up care to patients who underwent shunt surgery.
6. Provide follow-up care to patients who did not undergo shunt surgery.

(2) How do you examine patients with suspected iNPH? Please check all items that apply.

1. Perform no examination of patients with suspected iNPH.
2. Perform dementia screening tests such as assessment using Hasegawa Dementia Scale-Revised and Mini Mental State Examination.
3. Perform other cognitive tests.
4. Evaluate the triad symptoms of iNPH (i.e., cognitive impairment, gait disturbance, and urinary incontinence).
5. Perform brain computed tomography (CT).
6. Perform brain magnetic resonance imaging (MRI).
7. Perform other neuroimaging examinations.
8. Perform CSF examination or CSF tap test.

(3) Do you know and use the guidelines for the management of iNPH? Please check all items that apply.

1. I do not know the guidelines.
2. I know the guidelines but do not use them.
3. I use the first edition of the guidelines for the management of iNPH published by the Japanese Society of Normal-Pressure Hydrocephalus.
4. I use the second edition of the guidelines for the management of iNPH published by the Japanese Society of Normal-Pressure Hydrocephalus.
5. I am familiar with disproportionately enlarged subarachnoid space hydrocephalus (DESH) on brain MRI, which is described in the guidelines.
6. I use other guidelines for the management of iNPH.

(4) What are the difficulties in the evaluation and treatment of patients with suspected iNPH? Please check all items that apply.

1. Brain CT cannot be performed.
2. Brain MRI cannot be performed.
3. I am not confident in my assessment of DESH on brain MRI.
4. I have no experience or confidence to diagnose iNPH.
5. No physician who can perform lumbar puncture is available.
6. I have no knowledge of the methods or criteria for evaluating clinical symptoms in CSF tap test.
7. There is no hospital nearby where patients can be referred for detailed examination and treatment.
8. Patients are referred to neurosurgeons, but the neurosurgeons said the patients are not indicated for shunt surgery.

(5) To what extent do you think MCDs should evaluate and treat patients with cognitive impairment due to suspected iNPH? Please check the item that applies.

1. Refer the patient to specialized institutions when iNPH is suspected.
2. Perform detailed examination and diagnosis.
3. Provide treatment.
